# Interferon-γ-induced GBP1 is an inhibitor of human papillomavirus 18

**DOI:** 10.1186/s12905-024-03057-4

**Published:** 2024-04-15

**Authors:** Min Xu, Miao-Chun Lin, Zhao-Hui Li

**Affiliations:** 1 Laboratory of Prenatal Diagnosis, Mindong Hospital Affiliated to Fujian Medical University, Ningde, 355099 China; 2 Central laboratory, Mindong Hospital Affiliated to Fujian Medical University, Ningde, 355099 China

**Keywords:** Human papillomavirus 18, Guanylate-binding protein 1, Interferon gamma

## Abstract

**Background:**

Human papillomavirus (HPV) infection is an important factor leading to cervical cell abnormalities. 90% of cervical cancers are closely associated with persistent infection of high-risk HPV, with the highest correlation with HPV16 and 18. Currently available vaccines and antivirals have limited effectiveness and coverage. Guanylate binding protein 1 (GBP1) was induced by interferon gamma and involved in many important cellular processes such as clearance of various microbial pathogens. However, whether GBP1 can inhibit human papillomavirus infection is unclear.

**Results:**

In this study, we found that GBP1 can effectively degrade HPV18 E6, possibly through its GTPase activity or other pathways, and E6 protein degrades GBP1 through the ubiquitin-proteasome pathway to achieve immune escape.

**Conclusion:**

Therefore, GBP1 is an effector of IFN-γ anti-HPV activity. Our findings provided new insights into the treatment of HPV 18 infections.

## Introduction

Human papillomavirus (HPV) is a non-enveloped circular double-stranded DNA virus [1]. The genome can be divided into early protein coding region (E region), late protein coding region (L region) and long control region (LCR). More than 200 HPV types have been identified, including about 40 types that infect the mucosal epithelium. At least 12 types identified as carcinogenic (or high-risk) [2]. Globally, HPV16 and HPV18 cause about 70% of cervical cancers, as well as a higher proportion of other cancers attributed to HPV [3]. HPV6 and HPV11, which are not classified as carcinogenic, cause almost all anogenital warts and recurrent respiratory papillomatosis [4]. To prevent the most common HPV subtypes infection, the CDC recommends HPV vaccination for boys and girls starting at ages 11 to 12 [5].

Interferon-stimulated genes (ISGs) are a class of genes that are activated by interferons. Participate in immune regulation, inflammatory response, apoptosis and other biological processes, and play a crucial role in the process of host antiviral infection [6]. Wang Y et al. found that interferon stimulating gene SAMD4 family can inhibit HBV by directly binding to viral RNA. Li Z et al. concluded through experiments that APOBEC3G plays an anti-EV-71 role by inhibiting the activity of 5′-UTR [7, 8]. Guanylate-binding proteins (GBPs) belong to the GTPase family and the expression of GBPs can be induced by IFN-γ [6]. The GBP family mainly relies on its N-terminal GTPase active region and C-terminal isoprene or other means to exert antiviral effects. It has been found that GBP1 can inhibit vesicular stomatitis virus (VSV), swine fever virus (CSFV), cerebral myocarditis virus (EMCV) and so on through its GTPase activity [9]. In addition, GBP1 can inhibit dengue virus (DV) by regulating the transcriptional activity of NF-κB [10]. It has been reported that the expression of GBP1 in cervical cancer was significantly higher than that in normal cervical tissues (*p* < 0.01) [11], but whether GBP1 has anti-HPV effect and the related mechanism remain to be studied.

Our study found that the mRNA of HPV18 E6 in GBP1-transfected cells was not significantly different from that in the mock group. However, the protein of E6 was significantly lower than that of the control and the IFN-γ-induced GBP1 perhaps degrades HPV18 E6 protein through its GTPase. Importantly, in order to complete its own replication and survival, HPV18 E6 protein can degrade GBP1 through the ubiquitin-proteasome pathway to escape host inhibition. Our findings suggest that the interferon-γ-stimulating gene GBP1 has an anti-HPV effect, which provides new insights into the treatment of HPV infections.

## Materials and methods

### Cells and plasmids

HEK293T and Hela cells (ATCC, Rockefeller, MD, USA) were grown in DMEM (Gibco BRL, Gaithersburg, USA) containing 10% fetal bovine serum (FBS, Hyclone, Logan, USA), and 2 mmol/L L-glutamine (Amresco, Solon, USA), and antibiotics (10 µg/ml streptomycin and 100 IU/ml penicillin). Plasmids pcDNA3.1-EGFP, pcDNA3.1-RFP, pcDNA3.1-GBP1-EGFP and pcDNA3.1-E6-RFP were constructed by PCR.

### Transfection

Monolayer culture of HEK293T cells, grown overnight, transfected with different amounts of pcDNA3.1-GBP1-EGFP and pcDNA3.1-E6-RFP by Lipofectamine 2000 (Invitrogen, CA, USA). Simultaneously blank group and control group or mock group were set. The cells were incubated at 37℃ in a 5% CO2 incubator and observed at different times with inverted fluorescence microscope. Quantitative measurements were performed using multifunctional microplate reader (SpectraMax M5e, Molecular Devices, Sunnyvale, USA) with excitation and emission wavelengths of 479 and 517 nm (EGFP) and 580 and 620 nm (RFP), respectively. Expressed as relative fluorescence units (RFU) compare to the control group [12], and cells were harvested for Western blot described later.

### Quantitative real-time PCR

RT-qPCR was performed to determine HPV18 E6 mRNA after transfecting Hela cells. Briefly, RNA was isolated from infected cells with Trizol reagent (Invitrogen). The primers of oligo dT was used to reverse transcribe 1.5 µg RNA samples and at 70℃ 5 min, the cDNA was synthesized on ice for 5 min, and 5 min at 20℃, 42℃ for 1 h, then 15 min at 70℃, stored at 4℃. The qPCR was performed with SYBR green probe (Tiangen Biotech Co., Ltd. Beijing, China). The primers for the HPV18 E6 gene were as follows: forward primer, 5′-GCGCGCTTTGAGGATCCAACACGGCGA-3′; reverse primer, 5′-CTTGTGTTTCTCTGCGTCGTTGGAGTC-3′, and the primers for the GAPDH gene were as follows: forward primer, 5´-GGAGCGAGATCCCTCCAAA-3´; reverse primer, 5´´-GGCTGTTGTCATACTTCTCATGG-3´. The thermal cycling included 95℃ for 10 min, then 45 cycles of 95℃ for 15 s and 1 min at 60℃. The melting point analysis from 45℃ to 95℃ was used to verify the qPCR products and GAPDH was used as the reference gene to calculate relative expression.

### Protein quantification by western blot

Each group transfected cells were lysed in buffer (0.08 M Tris-HCl with 2.0% SDS, 10% glycerol, 0.1% bromophenol blue, and 0.1 M dithiothreitol) and heated at 100℃ for 30 min. Separated by SDS-PAGE with 12% acrylamide and electro-blotted on nitrocellulose paper at 120 V for 2 h. Blocked for 1 h at room temperature and incubated with the primary antibodies (anti-GBP1 mouse mAb、anti-HPV18 E6 mouse mAb and anti-GAPDH mouse mAb, respectively) at 4℃ overnight. Incubated with the HRP-goat Anti-Mouse IgG secondary antibodies for 1 h at 37℃. Then exposed in the darkroom with ECL chemiluminescence reagent kit and pictures were taken with Syngene Bio Imaging [13].

### Statistical analyses

Statistical analyses were performed with GraphPad Prism 8 (GraphPad Software, La Jolla, CA). Independent, two-sided Student’s t-test was used for comparison and three times experiments were repeated. *P* < 0.05 was considered statistically significant.

## Results

### GBP1 inhibits HPV replication

**Fig. 1:**
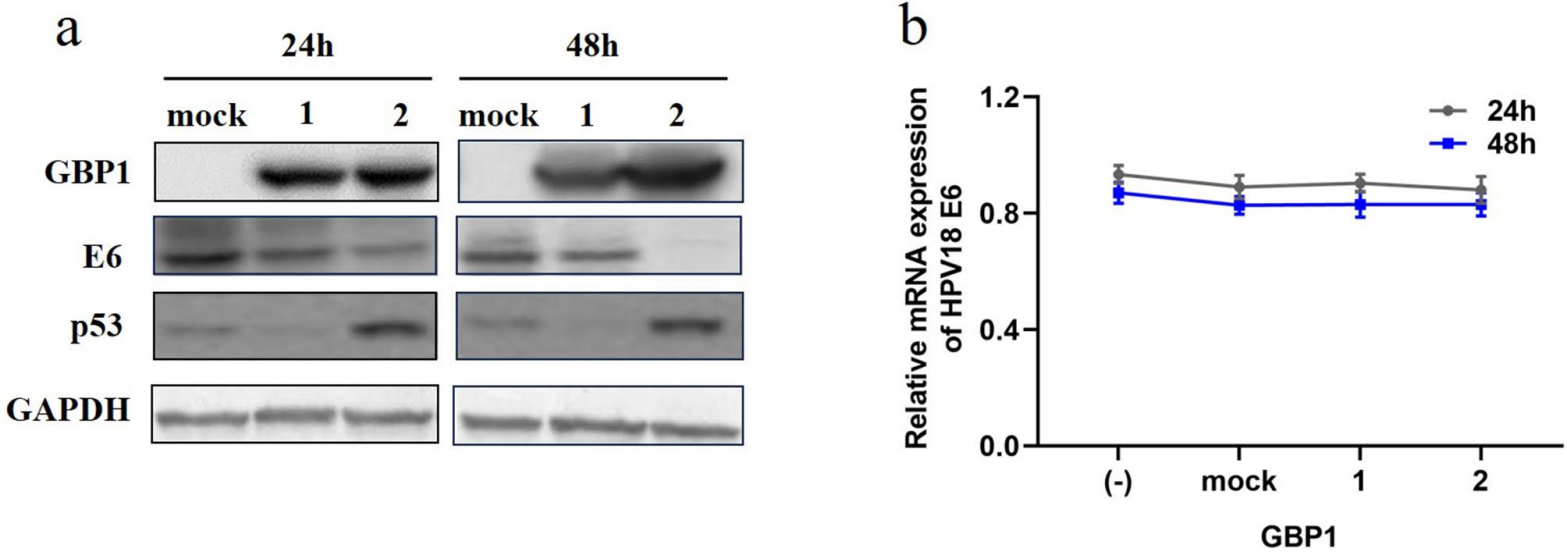
Expression levels of HPV protein and mRNA in Hela cells transfected with pcDNA3.1-GBP1-EGFP. (**a**) Western blot. (-) blank control group, (mock) 1.5 µg pcDNA3.1 vector was transfected, (1) 0.5 µg pcDNA3.1-GBP1-EGFP and 1 µg pcDNA3.1 vector were transfected, (2) 1.5 µg pcDNA3.1-GBP1-EGFP was transfected. (**b**) HPV18 E6 mRNA levels in Hela cells after transfected. Data were shown as mean ± SD. ∗ *P* < 0.05, ∗∗ *P* < 0.01

In order to prove that GBP1 has an anti-HPV effect. We transfected Hela cells with pcDNA3.1-GBP1-EGFP 0ug, 0.5ug and 1.5ug, respectively. Cells were collected 24 h and 48 h after transfection, the expression of HPV18 E6 were detected by Western Blot (Fig. 1a). The results showed that GBP1 significantly inhibited HPV replication at both 24 and 48 h after transfection. The expressin of HPV18 E6 protein was significantly reduced but mRNA level was not affected (Fig. 1b). This may suggest that GBP1 inhibits viral replication by down-regulating E6 at the protein level. At the same time, we found that the expression of p53 protein was up-regulated after GBP1 transfection.

### GBP1 down-regulates HPV E6 protein

**Fig. 2:**
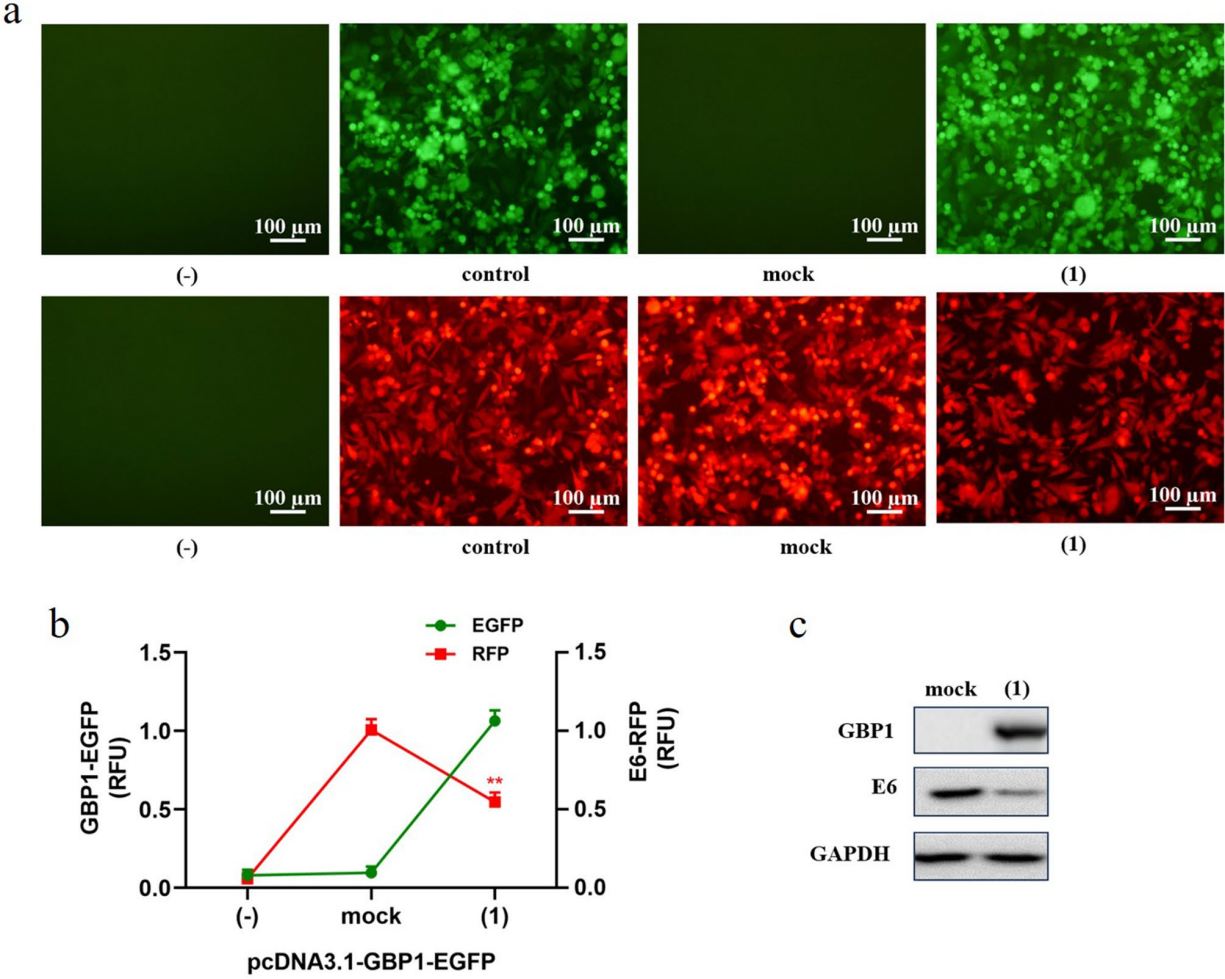
GBP1 inhibits HPV by degrading the E6 protein. Co-transfected pcDNA3.1-GBP1-EGFP and pcDNA3.1-E6-RFP into HEK293T cells in a 3:1 ratio. After 48 h transfected, the fluorescence signals from pcDNA3.1-GBP1-EGFP or pcDNA3.1-E6-RFP were observed under invert fluorescence microscope (**a**). The intensities of fluorescence from pcDNA3.1-GBP1-EGFP or pcDNA3.1-E6-RFP were detected by multifunctional microplate reader and expressed as relative fluorescence units (RFU) compare to the control group (**b**). (**c**) cells were harvested and the protein levels of GBP1 and E6 were determined by Western Blot using GAPDH as an internal reference. blank control (-), (mock) 1.5 µg pcDNA3.1 vector and 0.5 µg pcDNA3.1-E6-RFP were transfected, (control) 1.5µg pcDNA3.1 vector and 0.5µg pcDNA3.1-RFP or 1.5µg pcDNA3.1-EGFP and 0.5µg pcDNA3.1 vector were transfected, (1) 1.5 µg pcDNA3.1-GBP1-EGFP and 0.5 µg pcDNA3.1-E6-RFP were transfected. Data were shown as mean ± SD. ∗ *P* < 0.05, ∗∗ *P* < 0.01

We transfected pcDNA3.1-GBP1-EGFP and pcDNA3.1-E6-RFP into HEK293T cells at a ratio of 3:1. The fluorescence signals observed at different times from the encoded enhanced green fluorescence protein (EGFP) in pcDNA3.1-GBP1-EGFP, or from the encoded red fluorescent protein (RFP) in pcDNA3.1-E6-RFP under fluorescence microscope. The fluorescence signals (Fig. 2a-b) was significantly reduced when co-transfected with pcDNA3.1-GBP1-EGFP (*p* < 0.01). Cells were collected 48 h after transfection, the protein levels of GBP1 and HPV E6 were detected by Western Blot. GAPDH was used as an internal reference. As shown in Fig. 2c, we found that when GBP1 and E6 were co-transfected into HEK293T cells after 48 h, the level of HPV E6 protein was significantly decreased, indicating that GBP1 can degrade HPV E6 protein.

### Perhaps GBP1 down-regulates E6 protein through its GTPase activity

**Fig. 3:**
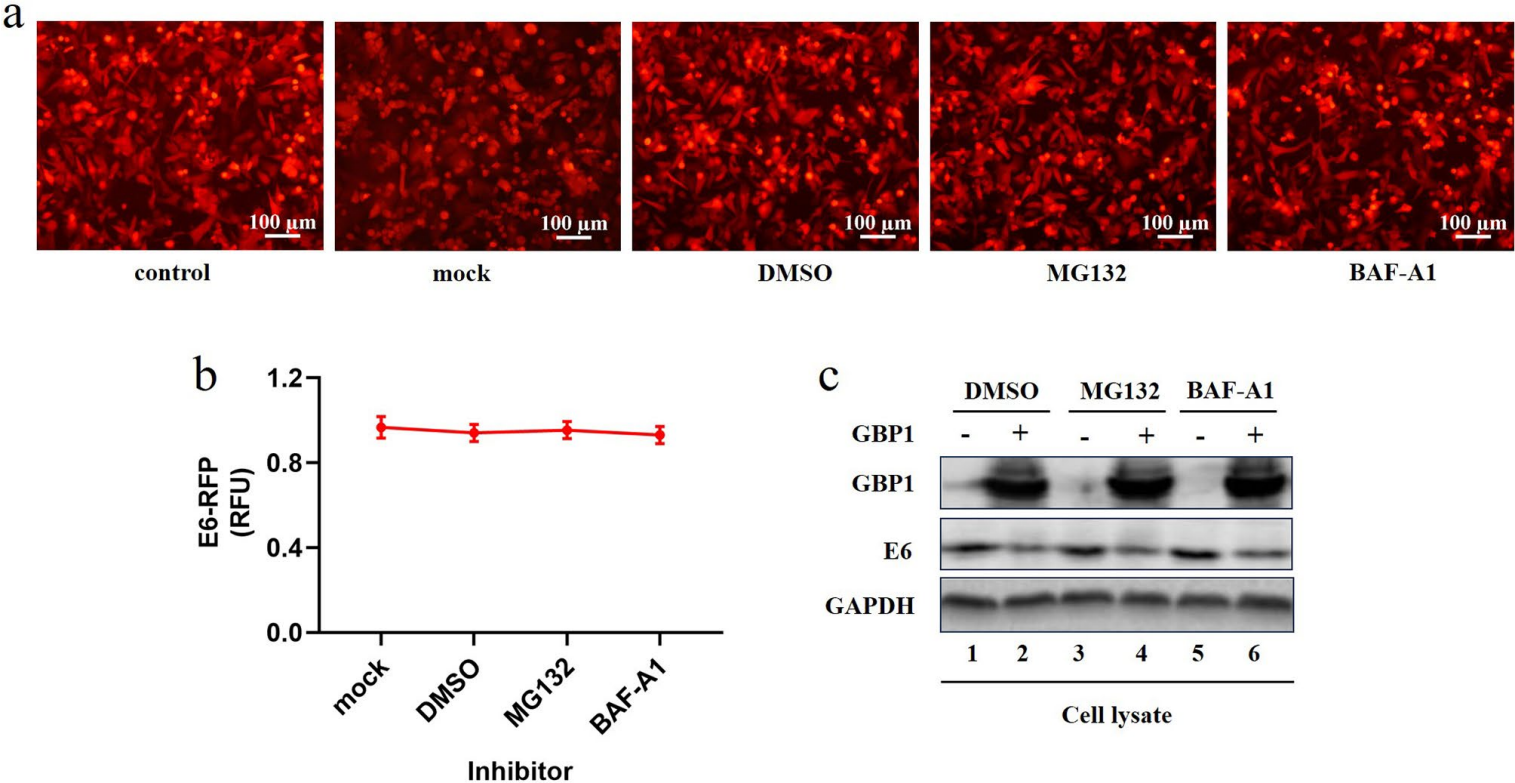
Identification of the relationship between GBP1 and E6. Using inhibitor to screen the way of GBP1 degrade HPV E6. Co-transfected pcDNA3.1-GBP1-EGFP and pcDNA3.1-E6-RFP into HEK293T cells at a 3:1 ratio, and the control group was transfected with pcDNA3.1-RFP or pcDNA3.1 vector (mock), and added the proteasome inhibitor Mg132 and autophagy inhibitor BAF-A1 at a ratio of 1:1000 after 36 h transfected respectively, DMSO was used as a control group. After 12 h of dosing, the fluorescence signals from pcDNA3.1-E6-RFP were observed under invert fluorescence microscope (**a**). The intensities of fluorescence from pcDNA3.1-E6-RFP were detected (**b**). (**c**) cells were harvested and the expression of GBP1 and E6 proteins were detected by Western Blot. (1,3,5) 1.5 µg pcDNA3.1 vector and 0.5 µg pcDNA3.1-E6-RFP were transfected. (2,4,6) 1.5 µg pcDNA3.1-GBP1-EGFP and 0.5 µg pcDNA3.1-E6-RFP were transfected. Data were shown as mean ± SD. ∗ *P* < 0.05, ∗∗ *P* < 0.01

In order to explore the specific way in which GBP1 down-regulates HPV E6 protein, pcDNA3.1-GBP1-EGFP and pcDNA3.1-E6-RFP were co-transfected into HEK293T cells at a ratio of 3:1. 36 h after transfection, Proteasome inhibitor MG132 and autophagy inhibitor BAF-A1 were added in the ratio of 1:1000, and DMSO was added in the control group. 12 h after administration, the fluorescence signals observed (Fig. 3a-b) and E6 protein level was detected by Western blot (Fig. 3c). The results showed that the fluorescence signal was not significantly reduced compared with the mock group and the inhibition of E6 by GBP1 cannot be restored by adding inhibitors Mg132 or BAF-A1, indicating that GBP1 may not degrade E6 protein through ubiquitin-proteasome or lysosome pathways. We hypothesized that GBP1 may degrade E6 through its GTPase activity or other pathways.

### HPV E6 protein degrades GBP1 through the ubiquitin-proteasome pathway

**Fig. 4:**
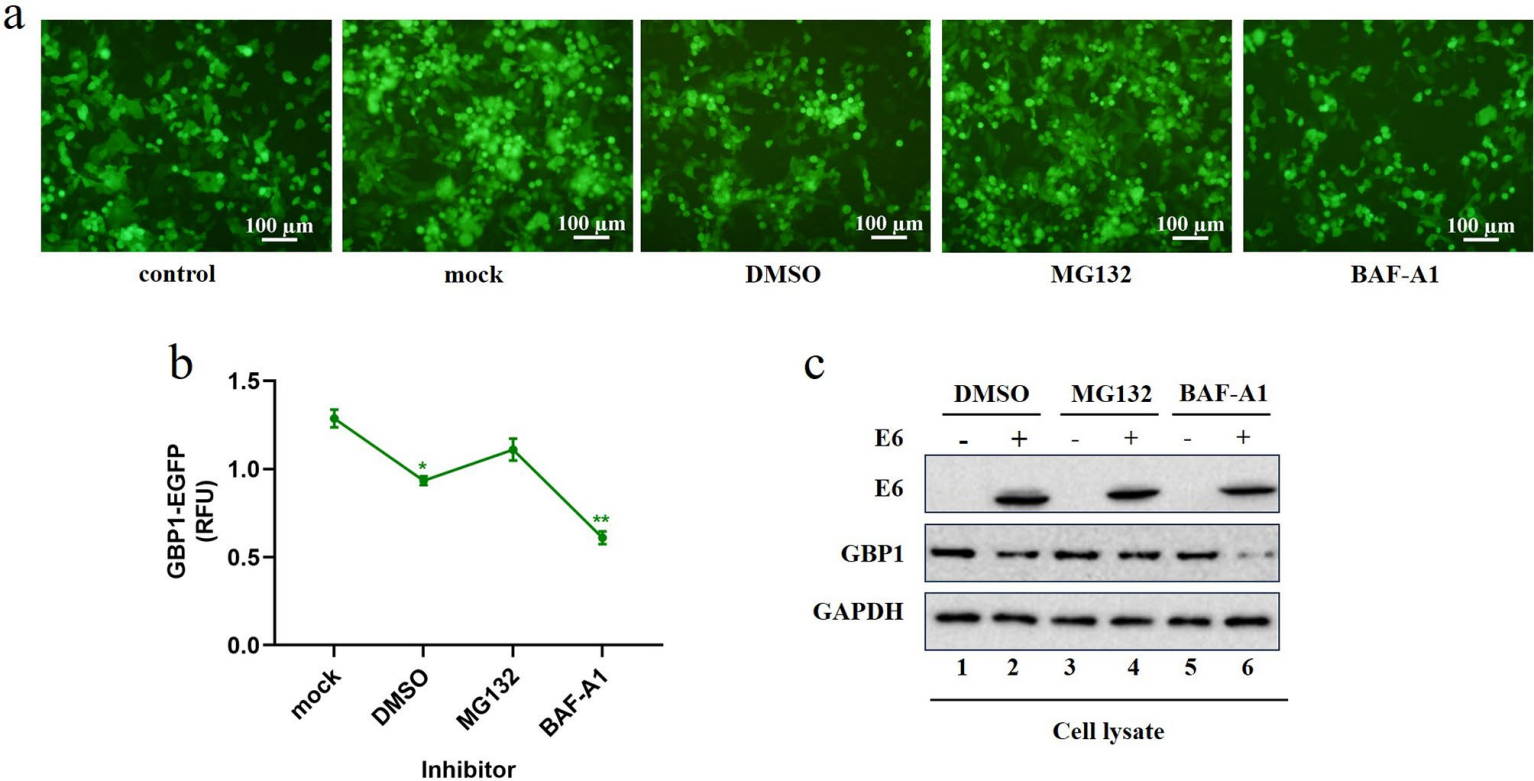
HPV E6 protein degrades GBP1 via the proteasome pathway. pcDNA3.1-GBP1-EGFP and pcDNA3.1-E6-RFP were co-transfected into HEK293T cells at a ratio of 1:3, and the control group was transfected with pcDNA3.1-EGFP or pcDNA3.1 vector (mock). 36 h after transfection, Mg132 (1:1000) and BAF-A1 (1:1000) were added, and the control group was added with an equal ratio of DMSO. After 48 h of transfection, the fluorescence signals from pcDNA3.1-GBP1-EGFP were observed (**a**). The intensities of fluorescence from pcDNA3.1-GBP1-EGFP were detected (**b**). (**c**) The expression of GBP1 and E6 protein was detected by Western Blot. (1,3,5) 1.5 µg pcDNA3.1 vector and 0.5 µg pcDNA3.1-GBP1-EGFP were transfected. (2,4,6) 1.5 µg pcDNA3.1-E6-RFP and 0.5 µg pcDNA3.1-GBP1-EGFP were transfected. Data were shown as mean ± SD. ∗ *P* < 0.05, ∗∗ *P* < 0.01

The way used by HPV E6 protein down-regulate GBP1 also with inhibitor screening. pcDNA3.1-GBP1-EGFP and pcDNA3.1-E6-RFP were transfected into HEK293T cells in a ratio of 1:3. 36 h after transfection, Mg132 and BAF-A1 were added proportionally. 48 h after transfection, the fluorescence signals were detected. As shown in Fig. 4a-b, compared with mock group, the fluorescence signal of DMSO (*p* < 0.05) and BAF-A1 (*p* < 0.01) groups were significantly decreased. In the DMSO group, E6 protein degraded GBP1, but in the proteasome inhibitor Mg132 group, we found that the inhibition of E6 on GBP1 was significantly weakened, and the expression of GBP1 was significantly restored, while this phenomenon was not found in the BAF-A1 group (Fig. 4c). Therefore, it is proved that E6 protein degrades GBP1 through the ubiquitin-proteasome pathway.

## Discussion

50-70% of women may have been infected with one type of HPV in their lifetime, and most HPV can be effectively cleared under the immune regulation of the body, but the infection of High-risk HPV (HR-HPV) was mostly directly related to High-grade squamous intraepithelial lesion (HSIL) [14]. HSIL is a common disease of female reproductive tract, which tends to be younger in recent years with symptoms of vaginal bleeding and some of them may progress to cervical cancer [15]. In addition, HR-HPV persistent infection is a key factor for recurrence of HSIL and cervical cancer and still occurred in some patients after loop electrosurgical excision procedure (LEEP) [16].

Studies have shown that proteins transcribed by ISGs can not only directly inhibit virus replication, but also play an indirect role in antiviral by regulating the expression of interferons (IFNs) [6]. Habiger C et al. found that IFN-κ inhibited viral transcription by inducing Sp100 protein expression in high-risk HPV-positive keratinocytes [17]. Huang X et al. found that activation of STING-TBK1 (TANK-binding kinase 1) promotes ubiquitin-proteasome degradation of the E7 oncoprotein, thereby inhibiting cervical cancer growth [18]. Our result shows that Interferon-γ-induced GBP1 is an inhibitor of human papillomavirus 18, after transfection with GBP1, HPV18 E6 mRNA level was not affected but the expression level of protein decreased significantly (Fig. 1a and b) and the expression of p53 protein was up-regulated while GBP1 degrading E6. HPV18 E6 inactivate the oncogene p53, leading to cell cycle dysregulation and neoplastic transformation of affected tissues [5]. Inhibitor screening indicate that GBP1 may degrade E6 through its GTPase activity or other pathways (Fig. 3a-c), which requires further research.

High-risk HPV must evade innate immune surveillance to establish persistent infection and expand the viral genome as it differentiates. Hong S et al. found that the innate immune regulator STAT-5 promotes HPV virus replication by activating the ATM DNA damage response [19]. Miyauchi, S et al. demonstrated that HPV E5 is a negative regulator of the interferon response pathway, antigen processing, and antigen presentation [20]. Reiser, J et al. suggested that HPV targets IFN-γ via different pathways in keratinocytes to inhibit antiviral ISGs and pathogen recognition receptors [21], and Castro-Munoz, L. J et al. demonstrated that the HR- (high risk) and LR- (low risk) HPV E1 proteins play an important role in suppressing antiviral immune responses [22]. Our results suggested that HPV18 E6 protein could degrade GBP1 through the ubiquitin-proteasome pathway to achieve immune escape (Fig. 4a-c). In summary, GBP1 is an effector of IFN-γ anti-HPV activity and our findings provided new insights into the treatment of HPV infections.

## Conclusion

After transfection with GBP1, the expression level of HPV E6 protein decreased significantly. In addition, inhibitor screening indicates that GBP1 may degrade E6 through its GTPase activity or other pathways, and E6 degrades GBP1 through the ubiquitin-proteasome pathway, which requires further research. Our findings showed that GBP1 is an effector of IFN-γ anti-HPV activity and provided new insights into the treatment of HPV infections.

## Data Availability

Datais provided within the manuscript, also, the datasets could be requested from the corresponding author upon reasonable request.
